# Neuroinflammation in Post-Traumatic Stress Disorder

**DOI:** 10.3390/biomedicines10050953

**Published:** 2022-04-20

**Authors:** Dong-Hun Lee, Ji-Young Lee, Dong-Yong Hong, Eun-Chae Lee, Sang-Won Park, Man-Ryul Lee, Jae-Sang Oh

**Affiliations:** 1 Department of Neurosurgery, College of Medicine, Soonchunhyang University, Cheonan Hospital, Cheonan 31151, Korea; madeby58@gmail.com (D.-H.L.); applesori82@gmail.com (J.-Y.L.); dydehdghd@gmail.com (D.-Y.H.); lec9589@gmail.com (E.-C.L.); ppphilio3@gmail.com (S.-W.P.); 2 Soonchunhyang Institute of Medi-Bio Science (SIMS), Soon Chun Hyang University, Cheonan 31151, Korea

**Keywords:** animal models, behavior test, damage-associated molecular patterns, hypothalamic-pituitary-adrenal axis, inflammation, kynurenine, neuroinflammation, post-traumatic stress disorder, serotonin, tryptophan

## Abstract

Post-traumatic stress disorder (PTSD) is a well-known mental illness, which is caused by various stressors, including memories of past physical assaults and psychological pressure. It is diagnosed as a mental and behavioral disorder, but increasing evidence is linking it to the immune system and inflammatory response. Studies on the relationship between inflammation and PTSD revealed that patients with PTSD had increased levels of inflammatory cytokine biomarkers, such as interleukin-1, interleukin-6, tumor necrosis factor-α, nuclear factor-κB, and C-reactive protein, compared with healthy controls. In addition, animal model experiments imitating PTSD patients suggested the role of inflammation in the pathogenesis and pathophysiology of PTSD. In this review, we summarize the definition of PTSD and its association with increased inflammation, its mechanisms, and future predictable diseases and treatment possibilities. We also discuss anti-inflammatory treatments to address inflammation in PTSD.

## 1. Introduction

Post-traumatic stress disorder (PTSD) is a mental illness caused by mental trauma following an accident involving mental shock, such as physical damage or anxiety [1]. According to the American Psychiatric Association, PTSD occurs in those who directly experience or witness the traumatic event or in close relatives or friends. Some of the major triggers of PTSD include violent crimes, accidents, emotional–social abuse, physical assault, military combat, civil unrest, natural disasters, and child abuse. PTSD is defined as a psychiatric disorder characterized by clinically significant social impairment, inability to work, or diminished mental ability to perform other daily functions [2]. PTSD is characterized by a psycho-emotional and neurophysiological spectrum, such as re-experiencing the traumatic events in the form of vivid memories, flashbacks, and nightmares [3]. The memories do not simply fade over time but may linger or even intensify for years [3]. These experiences are followed by overwhelming fear and strong physical sensations. Individuals with PTSD attempt to suppress memories or avoid activities reminiscent of the traumatic events by withdrawing from society. Excessive surrounding vigilance or response to unexpected noise demonstrates a state of fear as the individual either currently faces or recognizes a threat. These symptoms markedly impair personal, family, social, educational, occupational, and other important functional areas, diminishing quality of life [2,3].

The diagnostic symptoms of PTSD may include obsessive compulsion and depression, which are included in the PTSD checklist of the Diagnostic and Statistical Manual of Mental Disorders (fifth edition). People with PTSD can experience severe physical and emotional pain and dysfunction, which can persist for more than a month. Traditional cognitive theories of PTSD postulate that memory abnormalities manifested as dissociative flashbacks and intrusive memories are key contributors to the development and persistence of the disorder [4,5]. Individuals with PTSD may have fear of experiencing the trauma again, even in the absence of threats [6].

Currently, approximately 8% of the population are diagnosed with PTSD in the United States. Among adult Americans, the prevalence of PTSD is estimated to be 6.8%, according to the National Comorbidity Survey Replication [7]. The lifetime prevalence of PTSD is about 3.9% worldwide [8]. Lifetime PTSD prevalence rates following combat trauma may be especially high, ranging from 10.1% to 30.9% among United States veterans (Vietnam and subsequent conflicts) [9]. The public costs for PTSD treatment have been increasing by almost US 43 billion per year [3].

The Marine Resiliency Study reported that preexisting concentrations of C-reactive protein (CRP) were directly correlated with the occurrence and severity of PTSD, three months after a seven-month military deployment [10], and elevated levels of interleukin (IL)-6, IL-8, and transforming growth factor β (TGF-β) during hospitalization predicted the development of PTSD one month later [11]. These data suggest that immune dysregulation predisposes individuals to PTSD [12,13,14,15]. The elevation of inflammatory cytokines in PTSD has clinical significance, as chronic inflammation can adversely affect cardiovascular and physical health. In addition, individuals with PTSD are significantly more likely to suffer autoimmune disorders compared with those with other psychiatric conditions [16,17]. Inflammation-related mediators, such as IL-1, IL-6, and tumor necrosis factor alpha (TNF-α), may pass through the blood–brain barrier [18], and the overproduction of pro-inflammatory cytokines can activate brain microglia [19,20]. A few small studies have examined the cerebrospinal fluid levels of cytokines in PTSD but have yielded conflicting results [21,22].

Currently, only a few drugs are approved for PTSD treatment, and studies that determined the impact of immune abnormality treatment on improving PTSD symptoms are limited. [14,23,24]. Therefore, in this review, we present a detailed overview of the relationship and mechanisms that connect these two factors (with regard to neuroinflammation and PTSD) and review potential pharmacological treatments for PTSD treatment.

## 2. Pathophysiology of Inflammation in PTSD

### 2.1. Inflammation and Disease

Inflammation is the body’s complex biological response to pathogens, damaged cells, and harmful stimuli [25] and includes immune cells, blood vessels, and molecular mediators. It is a protective response that helps eliminate the cause of cell damage and remove and repair damaged cells and tissues. The clinical signs of inflammation include fever, pain, erythema, edema, and loss of function. Inflammation is caused by various factors, including physical causes such as burns, frostbite, and other physical injuries; immune reactions to infection and hypersensitivity reactions to pathogens; and chemical causes, such as chemical stimulators or alcohol. These factors can trigger acute inflammation lasting several days, or chronic inflammation, lasting months to years.

Acute inflammation occurs immediately after injury and is caused by pathogens, allergens, and toxins. Cytokines and chemokines bring neutrophils and macrophages to inflammatory sites. Acute inflammation usually begins with macrophages and dendritic cells. These cells possess receptors known as pattern recognition receptors (PRRs), which recognize and bind sub-molecules of pathogen-associated molecular patterns (PAMPs) and damage-associated molecular patterns (DAMPs). When PRRs are activated from infection or other damage, they release inflammatory mediators. This causes fever and vasodilation, which promotes local redness due to increased blood flow. Blood vessel permeability increases, resulting in plasma protein and body fluid exudation into the surrounding tissues, and mediated molecules help neutrophilic and macrophagic tissue infiltration.

Chronic inflammation can last for months to years. It can also be promoted by daily metabolic imbalances such as obesity, smoking, and a non-nutritious diet, that can later develop into diabetes, cardiovascular diseases, allergies, and chronic obstructive pulmonic disease.

### 2.2. Neuroinflammation and Glial Cells

Neuroinflammation occurs when inflammatory reactions affect the nerve tissues. The brain was once considered an immune-privileged site except in certain cases of illness and injury, but it is now well known that peripheral pro-inflammatory cytokines can affect the brain through several mechanisms, including by crossing the blood–brain barrier [26]. Inflammation in the central nervous system and surrounding areas can contribute to neuroinflammation through the activation of microglia and astrocytes. Neuroinflammation, unlike acute inflammation of the central nervous system, is considered a type of chronic inflammation. Acute inflammation of the central nervous system occurs immediately after damage, causing reactions such as activation of the inflammatory molecules, endothelial cells, and tissue edema. On the other hand, neuroinflammation is when nerve cells are continuously activated, and other immune cells are mobilized to the brain. It is closely related to neurodegenerative diseases. IL-1β, IL-6, and TNF-α are reported to affect the brain at its cognitive, functional, and morphological levels, as well as neurogenesis, synaptic plasticity, and memory learning [27].

Astrocytes are the most abundant glial cells in the brain. They are involved in the maintenance and support of neurons and constitute an important element of the blood–brain barrier. When a neuron is damaged by brain injury, the microglia are activated, and astrocytes detect the signal emitted. Various factors are then released for axon regeneration. Microglia are innate immune cells of the central nervous system, but they can lose function due to disease [28]. Microglia exposed to disease and stress persist in its activation patterns [29] and can overproduce cytotoxic molecules, such as anti-inflammatory cytokines [30]. Diseases of microglia and astrocytes can lead to functional and structural brain changes, as well as behavioral modifications, related to PTSD. Neuroinflammation is also associated with an increased tendency toward aging and neurodegenerative diseases.

## 3. Increased Pro-Inflammatory and Anti-Inflammatory Markers in PTSD

Cytokines are small proteins that are important for cell signaling. They can move across the cellular lipid bilayer and are involved in autocrine and endocrine signals as immunomodulators, albeit being different from hormones. Chemokines, interferons, interleukins, and lymphokines are all included in the cytokine family and are produced in a wide range of cells, including endothelial cells and fibroblasts, macrophages, B lymphocytes, T lymphocytes, and mast cells. Cytokines work through receptors on the cell surface and are particularly important immune mediators. IL-1, IL-6, IL-18, TNF-α, interferon gamma (IFN-γ), and granulocyte–macrophage colony-stimulating factor (G-CSF) are examples of cytokines that mediate the inflammatory response. Conversely, cytokines that lower the inflammatory response include IL-4 and IL-10.

PTSD is associated with dysregulation of the immune response, which is reflected by an increase in pro-inflammatory cytokines, such as IL-6 and IL-17, and a decrease in anti-inflammatory cytokines, including IL-4 [31,32,33]. Patients with PTSD are reported to have significantly higher blood levels of inflammatory markers, such as IL-1β, IL-6, TNF-α, and C reactive protein (CRP), compared to healthy controls (Table 1) [34,35,36,37,38,39,40,41].

CRP is also a protein that responds to inflammation. It is an acute protein that increases with the secretion of interleukin-6 and is used as an inflammatory marker by promoting apoptosis through complement systems and bacterial action by macrophages. One study revealed that plasma CRP levels in veterans are potentially associated with the onset of PTSD symptoms, suggesting that inflammation may be a risk factor for PTSD [10,12,13,32,42,43,44,45,46].

One of the largest studies exclusively of men exposed to combat trauma reported a significantly elevated pro-inflammatory composite, comprising IL-1β, IL-6, TNF-α, IFN-γ, and CRP levels in those with PTSD compared to those without PTSD [47]. The predominant cytokines were different for each group, and differences were observed in TNF-α, IFN-γ, and IL-6 values. The pro-inflammatory cytokine levels remained significantly higher in individuals with PTSD after controlling for early-life trauma, major depressive disorder and its severity, body mass index, ethnicity, education, asthma and/or allergies, time since combat, potentially confounding inflammatory illnesses, and medications. Immune activation of PTSD was also found to be significantly high in male groups who experienced combat trauma [35].

Conversely, several other studies have reported no significant difference in inflammatory marker levels between patients with PTSD and healthy controls [34,35,48,49].

## 4. Animal Models of PTSD

### 4.1. Development of Animal Models of PTSD for Animals

Stress exposure in primates showed similar results to humans [50,51,52,53,54]. In addition, various animal models have been developed to replicate the characteristics of PTSD. These models were developed using physical, social, and psychological stressors, and several factors can be applied to animals at the same time [55,56,57].

#### 4.1.1. By Physical Stressors

**Figure 1:**
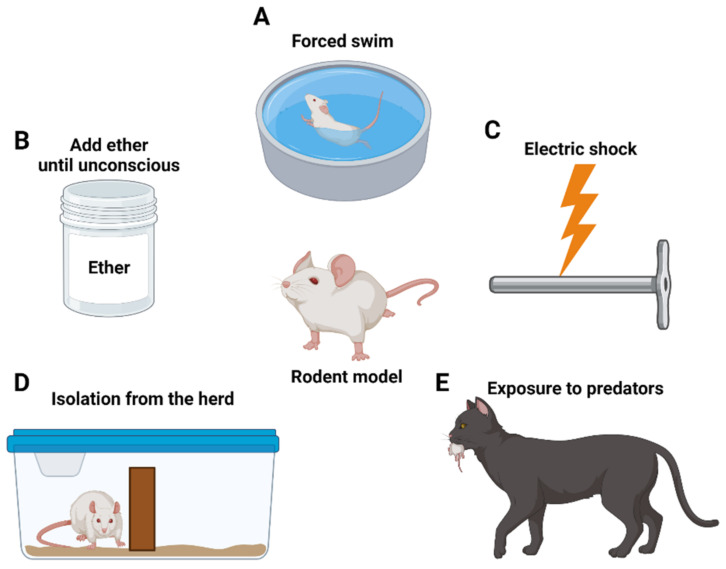
Development of PTSD animal models. (**A**) Forced swimming; (**B**) ether is applied to the rodent model until it becomes unconscious; (**C**) electric shock through metal bar; (**D**) isolation from the parent herd; and (**E**) exposure to predators.

Physical stressors induce anxiety via direct physical stimuli that the target tries to avoid. One can say that they are similar to a combat soldier’s accident or a PTSD patient who has experienced violence. The first PTSD research method involves restraining rats for 2 h, followed by forced swimming (Figure 1A) for 20 min, and then exposing them to ether until they become unconscious, after 15 min (Figure 1B). This model increases the negative feedback of the hypothalamic–pituitary–adrenal (HPA) axis [58,59]. Second, the foot shock method uses metal bars to transmit electrical shocks to the animals’ feet (Figure 1C) [60]. At the same time, an auditory signal is relayed, and later on the same sound is used to induce post-impact horror recollection [61]. Repeated stress exposure increased anxiety behavior in the Elevated Plus maze [62], characterized by elevated cortisol levels and increased negative feedback of the HPA axis [63].

#### 4.1.2. By Social Stressors

Social stressors are not direct stimuli, but instead occur in basic social animal structures. This is similar to PTSD in humans resulting from instances such as bullying. The first approach is the housing instability method, in which individual animals are paired with different cage groups daily, often exposing them to the smell of predators [64]. Rats exhibit increased corticosterone inhibition and HPA dysfunction in this setting [65]. Second, social instability and early-life stress methods include isolation of adult mice from the herd [66] or isolation from the mother (Figure 1D). Separating mothers from babies mirrors childhood trauma in humans, and it leads to anxiety-like behavior and sexual dependence mediated by HPA functions [67].

#### 4.1.3. By Psychological Stressors

Psychological stressors consider that most human cases of PTSD result from vulnerability to trauma. Animal models that are approached directly or indirectly by natural predators experience chronic social instability. Predator-based psychosocial stress (PPS) methods periodically prevent animal movement, introduce direct confrontation with the naturally feared predators, and induce a great amount of stress (Figure 1E). The PPS model mimics the reduction of glucocorticoids [65,68]. Similarly, predator scent stress method indirectly causes stress on the animals through the release of the predator’s scent, without direct exposure to the predators [69].

### 4.2. Inflammatory Changes in Animal Models

Inflammatory responses in animal models were evident in specific brain regions. A predator-exposure rat study found increased pro-inflammatory cytokines in the hippocampus, amygdala, and prefrontal cortex, with a concomitant reduction in anti-inflammatory cytokines [70,71]. Similarly, a stress-enhanced fear-learning model revealed increased hippocampal IL-1β concentrations, and learning decrement was prevented by blocking central IL-1β signaling after the stressful stimulus [72]. In a predator scent-stress mouse model, activation of the nuclear factor k light-chain enhancer pathway of activated B cells (NF-κB) promoted anxiety, and inhibition of this pathway reduced both IL-1β concentrations and anxiety levels [73].

Molecular investigations revealed a relationship between neuroinflammation and behavioral manifestations of simulated PTSD in rodents [74]. Pro-inflammatory mediators were upregulated in the brain immediately and up to four weeks after stress withdrawal. These cytokines inhibited neurogenesis [74].

In addition, rodents showed lower plasma norepinephrine [75,76] and higher plasma corticosterone concentrations during stress [77,78,79].

In animal studies, inflammation due to social stress is well described; social stress increases the number of macrophages and stimulates the release of plasma cytokines [80,81,82]. A study of the “social defeat test” using a rat model confirmed that IL-6 levels increased within an hour after exposure to stress due to defeat and returned to normal within 24 h [83]. In addition, MCP-1 (Monocyte Chemoattractant Protein-1), an anti-inflammatory chemokine, increased significantly after 24 h [83]. These research findings indicate that stress causes inflammation, but this response may be temporary and may have different time-dependent outcomes.

## 5. Mechanisms of Neuroinflammation in PTSD

### 5.1. DAMPs Pathway

The innate immune response is thought to be a major cause of many mental disorders, including PTSD [84,85]. In contrast to previous beliefs, stress experience is now known to be associated with neuroinflammation, and clinical reports have shown that stressed individuals display high inflammatory disorders, leading to a sharp increase in pro-inflammatory cytokines in stress-responsive areas of the brain, such as the hypothalamus and hippocampus [26,86,87]. Damage-associated molecular patterns (DAMPs) are host-derived non-bacterial immune response molecules released due to PTSD-related stress. These molecules act as danger signals when cells are damaged. When DAMPs are released from cells, pattern recognition receptors (PRRs) are expressed in astrocytes and microglia to promote neuroinflammatory reactions. The PRR is a protein expressed by innate immune system cells such as dendritic cells, macrophages, monocytes, and neutrophils, and is a receptor that recognizes DAMPs and plays an important role in innate immunity [88]. One of the PRRs, the Toll-like receptor (TLR), is expressed in various cells of the CNS, including neurons, microglia, and astrocytes [89]. These PRRs are activated by detecting endogenous molecules such as DAMPs, and the persistent neuroinflammatory action of glial cells can lead to neurodegeneration [90,91].

### 5.2. HPA Axis Pathway

Previous studies have argued for an interaction between the HPA axis and inflammatory cytokine regulation. The pro-inflammatory state of PTSD arises from stress responses associated with changes in the HPA axis and autonomic nervous system activity [92,93]. The HPA axis is a neuroendocrine system that plays a pivotal role in maintaining the stress response and homeostasis.

**Figure 2:**
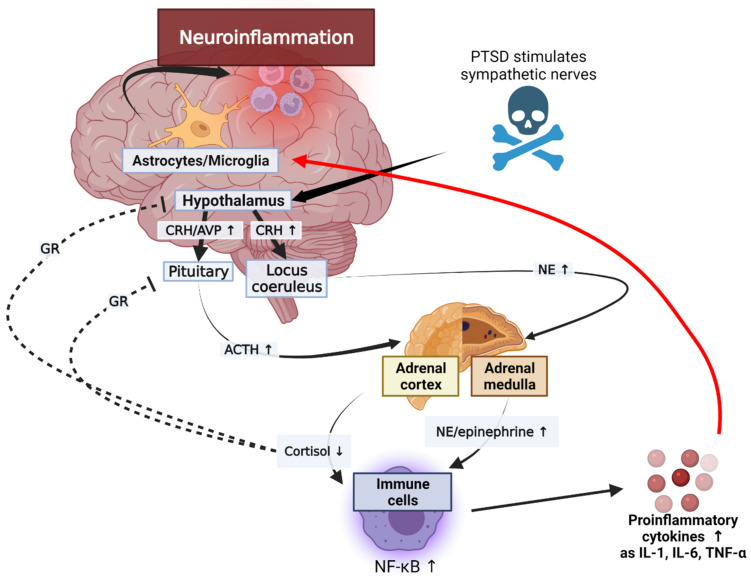
Mechanisms of increased inflammation in PTSD. The immune system interacts with the hypothalamus, pituitary, and adrenal axis. The black arrow represents a series of sequential processes, the red arrow represents the final result of neuroinflammation, and the dotted line represents inhibition according to the process. ACTH, adrenocorticotropin; AVP, arginine vasopressin; CRH, corticotropin-releasing hormone; GR, glucocorticoid receptor; NE, norepinephrine; NF-κB, nuclear factor-κB; IL, interleukin; TNF-α, tumor necrosis factor α.

The secretion of glucocorticoids in the adrenal cortex increases in response to various stressors. The production of glucocorticoids is self-regulated by negative feedback through the glucocorticoid receiver of hypothalamus and pituitary gland [94]. These stress-inducing responses increase inflammation through the following scenario (Figure 2).

**Figure 3:**
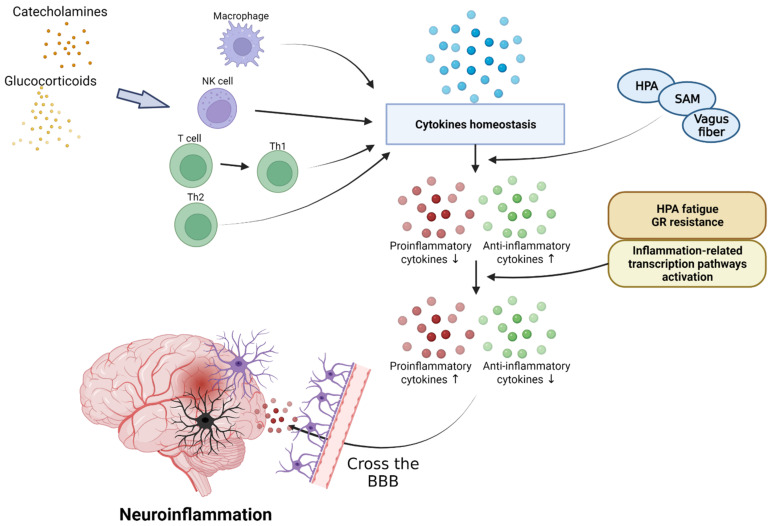
Effect of inflammatory cytokines in PTSD. PTSD interferes with cytokine homeostasis and inhibits the secretion of anti-inflammatory cytokines through the activation of the HPA axis, SAM axis, and vagus nerves. Continuous PTSD can induce HPA fatigue and increase glucocorticoid receptors, inducing inflammatory reactions and causing neuroinflammation by crossing the BBB. HPA, hypothalamic–pituitary–adrenal; SAM, sympathetic–adrenal–medullary system.

Stressors activate the HPA axis through stimulation of the sympathetic nerve of the autonomic nervous system. Epinephrine and norepinephrine (NE) are released by the activated splanchnic nerves. An activated hypothalamus promotes the secretion of corticotrophin-releasing hormone (CRH) and arginine vasopressin (AVP) [95]. CRH stimulates the sympathetic nervous system (SNS) to produce catecholamines, including NE. NE secreted by adrenal medulla upregulates NF-κB. Cortisol inhibits the NF-κB signal and reduces the synthesis and release of inflammatory cytokines by mediating apoptosis regulation. Adrenal cortex is stimulated by the hypothalamus-pituitary axis; it increases and decreases cortisol levels in normal humans and patients with PTSD, respectively. Glucocorticoid secretions through the HPA axis typically inhibit lymphocyte proliferation and reduce the secretion of pro-inflammatory cytokines, including IL-6, IL-12, IFN-γ, and TNF-α, during stress conditions [96]. However, the reduced activity of cortisol in PTSD can exacerbate inflammation [97]. This reduction in cortisol activity is mediated by 11B-HSD. Inactive cortisone is metabolized to active cortisol by 11B-HSD1, and cortisol is converted to the inactive form by 11B-HSD2. These corticosteroid metabolites change significantly in inflammatory conditions. In patients with inflammatory diseases, the expression of 11B-HSD1 increases and stress also increases the macrophage-related expression of 11B-HSD1. In addition, inflammation increases the activity of 11B-HSD2, leading to lower cortisol levels in inflammatory areas in the kidneys, brain, and adrenal glands of animals with PTSD. Glucocorticoid signaling is mediated by the glucocorticoid receptors (GR) [97]. T cells in patients with PTSD showed reduced glucocorticoid sensitivity. In addition, the decrease in glucocorticoid sensitivity due to the action of inflammatory states or inflammatory cytokines was demonstrated in an in vitro study [98]. These results suggest that the GR resistance can be induced during inflammatory reactions, and that the immune system can be an additional pathway to affect the HPA axis (Figure 3). Further, since the GR isoforms levels may change in inflammatory conditions, studies on the isoforms may also be used to better explain the glucocorticoid resistance [97]. This upregulation of NE and the reduction in cortisol levels and GR activity can increase the expression of pro-inflammatory proteins by NF-κB, leading to inflammatory responses in patients with PTSD. These interleukins can be transported from the blood to the brain via a blood–brain barrier (BBB), and the capillaries lacking a BBB allow cytokines to access the preoptic area of the hypothalamus directly. Cytokines acting on CNS through endothelial cells of a BBB contribute to neuroinflammation. In addition, when the immune system is strongly activated by PTSD, glial cells and other brain immune cells change form and secrete high levels of inflammatory cytokines and prostaglandins, which are involved in inflammation through vasodilation [99]. IL-1 receptors (IL-1Rs) are produced by microglia, astrocytes, and brain oligodendrocytes, and have been shown to be highly concentrated in the pituitary and meninges [100]. IL-1R mRNA is expressed in the capillaries and glial cells throughout the hippocampus, choroid plexus, cerebellum, and brain, and the IL-1 type 1 receptor is expressed in humans’ hypothalamus [101,102]. The secreted inflammatory cytokines are received in these glial cells and contribute to neuroinflammation. Therefore, the impairment of the HPA axis due to PTSD can lead to neuroinflammation.

### 5.3. Serotonin and Tryptophan–Kynurenine Pathway

**Figure 4:**
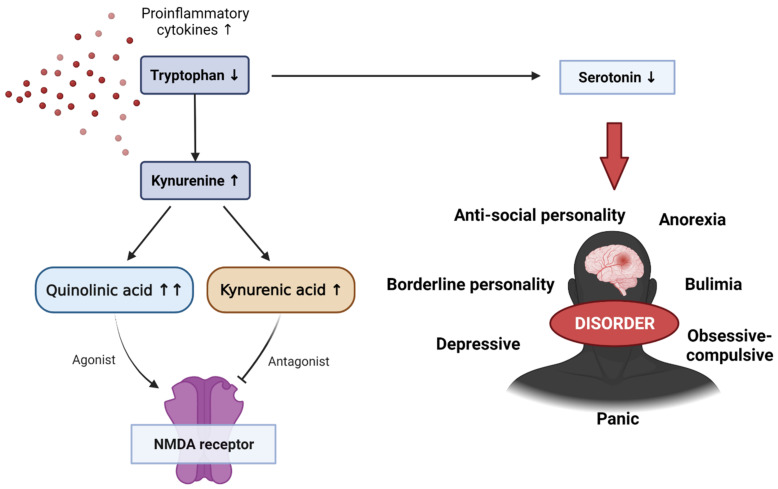
The effect of reduced tryptophan on the kynurenine pathway, as well as various emotional–behavioral disorders caused by a decrease in serotonin levels due to reduced tryptophan. NMDA, *N*-methyl-d-aspartate.

Tryptophan is an essential amino acid used in protein biosynthesis and is mainly metabolized with kynurenine. Kynurenine is a metabolite of tryptophan that performs a variety of biological functions, including vasodilation and immune response during inflammation. This kynurenine pathway is modified in several diseases, including mental disorders such as schizophrenia and depressive disorders. Pro-inflammatory cytokines can enhance the activity of indolamine 2,3-dioxigenase (IDO), the first and rate-limiting enzyme in the tryptophan degradation pathway. Elevated levels of pro-inflammatory cytokines increase IDO enzyme activity. IDO is involved in tryptophan metabolism, which upregulates the production of kynurenine. Kynurenine is converted into several metabolites, including quinolinic and kynurenic acids, and it is involved in the activity and inhibition of NMDA (*N*-methyl-d-aspartate), with elevated pro-inflammatory cytokines producing relatively larger amounts of quinolinic acid (Figure 4) [103,104]. This increase in quinoline acid has a potent neurotoxic effect, which can activate microglia and macrophages, increase reactive oxygen and reactive nitrogen species due to lipid peroxidation, and interfere with nerve function or cause apoptosis. Some studies have also shown that quinoline acid may also be linked to mental disorders, although the mechanism is still unclear. However, quinoline acid has been found in the postmortem brain of depressed patients.

Serotonin is a neurotransmitter that regulates many physiological processes such as mood, cognition, reward, learning, memory, and vasoconstriction. It is derived from the decarboxylation of tryptophan and is involved in controlling social behavior and interactions, as well as response to stimuli. Therefore, abnormal serotonin levels can lead to emotional and behavioral symptoms and disorders such as cognitive impairment, depression, suicidal thoughts, anorexia, antisocial and borderline personality disorders, bulimia, obsessive compulsive disorder, and panic disorders.

## 6. Potential Treatment in PTSD

As mentioned earlier, PTSD and inflammation appear to be closely related. However, pharmacological treatments for PTSD are not diverse. Therefore, the development of new drug therapies for PTSD based on the biological mechanisms of inflammation is of great interest. Several existing types of anti-inflammatory drugs are summarized in Table 2.

Animal studies have investigated the efficacy of anti-inflammatory therapies in the modification of PTSD-like features [105,106,107,108,109,110]. In a rat model of psychogenic stress with elevated cytokine levels, treatment with minocycline, an anti-inflammatory, antiapoptotic, and neuroprotective tetracycline antibiotic, reduced levels of IL-1, IL-6, and TNF-α in the hippocampus, frontal cortex, and hypothalamus, and alleviated anxious behaviors [109]. In another model with increased inflammation, ibuprofen not only decreased hippocampal expression of the pro-inflammatory mediators TNF-α, IL-1b, and brain-derived neurotrophic factor (BDNF), but also alleviated anxiety symptoms [108]. In a mouse foot-shock fear-conditioning study, treatment with cyclooxygenase-2 inhibitors reduced a variety of stress-induced behavioral pathologies [105]. Selective serotonin reuptake inhibitors, including fluoxetine, are considered first-line medications for human PTSD. In the foot-shock fear-conditioning mouse model [107], administration of fluoxetine improved PTSD symptoms and concurrently inhibited stress-induced inflammatory gene expression [105,111].

Angiotensin-converting enzyme inhibitors and angiotensin receptor blockers, which are mainly used to treat hypertension, have been proposed as anti-inflammatory treatments [112,113]. A rat study with PTSD caused by noise and light electric shock showed that ARBs helped alleviate fear [114].

Cannabis is a psychotropic substance that has anti-inflammatory properties. Endocannabinoids appear to be involved in several aspects of PTSD, including inflammation, and very few studies have reported the potential usefulness of cannabis in the treatment of PTSD. However, cannabis poses a risk of addiction or drug abuse in mental illness.

Low cortisol levels were found in PTSD patients, and the treatment effects of exogenous glucocorticoids were investigated. They showed favorable results, alleviating PTSD symptoms when administered alone [115] and with psychotherapy [116,117].

Treatment with monoclonal antibodies is a simple way to block inflammatory cytokines. Although not yet tested in PTSD patients, the inhibition of increased inflammation due to cytokine blocking may be beneficial.

Non-steroidal anti-inflammatory drugs inhibit COX-1 and-2, which are involved in cytokine production and, thus, exhibit anti-inflammatory effects. Although non-steroidal anti-inflammatory drugs have not been used in PTSD patients, studies using the PTSD rat models showed decreased inflammatory cytokine levels and behavioral symptoms [108].

Another novel treatment approach is to understand the correlation of neural oscillatory mechanisms, which can alleviate fear and anxiety symptoms [118]. The electrical signal changes in neurons include alpha (8–12 Hz), beta (12 Hz), delta (0.5–4 Hz), theta (4–8 Hz), and gamma (>25 Hz) waves. Electroencephalogram (EGG) studies have reported increases in delta/theta wave (related to arousal conditions and heavy exercise) strength in prefrontal, frontal, and midline channels in fear from conditional and unconditional stimuli. In addition, a decrease in alpha/beta wave (related to rest and alertness, active thinking, and concentration) strength was observed in the parietal and occipital channels. Deep brain stimulation (DBS) is a nerve stimulation device that sends electrical impulses through implanted electrodes, which can mitigate fear and anxiety symptoms by regulating neural oscillatory activity in the human subthalamic nucleus (STN) and animal dorsomedial ventral striatum (dmVS). Vagal nerve stimulation (VNS), another neuroregulatory approach, may also eliminate PTSD-like symptoms such as anxiety and social avoidance one week after treatment [119].

In addition, potential beneficial effects of health promotion behaviors, such as exercise and diet, have been reported in patients with PTSD. These interventions can be used as an alternative treatment for PTSD as they impose less side effects and are less costly.

## 7. Conclusions

Various studies have suggested that inflammation may play an important role in the pathophysiology of PTSD, providing evidence of non-infectious inflammation caused by interactions between the immune system and the brain mediated by psychological stress. These findings in clinical and animal studies show that PTSD can be associated with systemic and CNS inflammation and is not a mere isolated psychological response.

In addition to the immune system, regulatory disorders in other stress-response systems, including the HPA axis and the SNS, have been shown to be involved in the pathophysiology of PTSD. There are complex interactions between these systems, and the confusion of these interactions can play an important role in the pathogenesis of PTSD. However, studies on the inflammation of PTSD and its association with HPA axis dysfunction are still limited. Further studies must be conducted to clarify the underlying mechanism. Furthermore, genetic factors, health-related factors, and transcriptional factors should be considered in addition to environmental and lifestyle factors that increase or decrease inflammatory responses. It is also recommended to further study the underlying mechanisms of the relationships between the GR, PTSD, and inflammation.

Animal studies of PTSD have also shown that behavioral changes (reflecting fear memory) are associated with immune dysfunction and inflammation [105,106]. In terms of treatment strategies, it may seem premature to talk about the possibility of future inflammatory drug targets before the relationship between inflammation and PTSD has been completely clarified. In conclusion, this review has summarized the relationship between inflammation and PTSD and has suggested anti-inflammatory treatment as a good strategy for the management of patients with PTSD. Parallel to continuous epidemiological investigations, delicate mechanistic studies are needed to find new treatment options.

## Figures and Tables

**Table 1:** Comparison of pro-inflammatory and anti-inflammatory markers between healthy controls and PTSD patients.

Marker	Cytokine (Human)	Healthy Controls (*n*)	PTSD Patients (*n*)	Healthy Controls	PTSD Patients	*p*-Value	References
Pro-inflammatory marker	IL-6	65	40	0.81 (0.60–0.95) pg/mL	0.95 (0.70–1.38) pg/mL	0.009	[34]
	IL-6	33	33	15.15 (12.31–21.88) pg/mL	19.57 (13.13–30.87) pg/mL	0.002	[36]
	CRP	355	229	2.53 (±5.94) mg/L	2.85 (±3.67) mg/L	0.004	[40]
	CRP	123	163	0.173 mg/dL	0.257 mg/dL	0.004	[41]
Anti-inflammatory marker	IL-10	33	33	24.16 (1.74–39.32) pg/mL	55.67 (31.21–78.27) pg/mL	0.002	[36]

CRP, C-reactive protein; IL, interleukin; PTSD, post-traumatic stress disorder.

**Table 2:** Anti-inflammatory drugs that are potentially effective in treating PTSD.

[33]	Typical Drugs	Anti-Inflammatory Mechanism
ACE inhibitors and ARBs	Captopril (ACE inhibitors) Candesartan (ARBs) Telmisartan (ARBs)	Prevents the synthesis of ACE inhibitors or blocks receptors of ARB angiotensin II that increase inflammation
Cannabis	Nabilone	Elevates endocannabinoid signaling that has anti-inflammatory effects
Glucocorticoids	Hydrocortisone, Prednisolone, Dexamethasone	Inhibits the expression of cytokines by a combination of genomic mechanisms
Monoclonal antibodies against cytokines	Infliximab (anti-TNF-α), Adalimumab (anti-TNF-α), Tocilizumab (anti-IL-6 receptor)	Prevents cytokines from binding to their receptors
Non-steroidal anti-inflammatory drugs	Celecoxib, Ibuprofen, Naproxen	Reduces pro-inflammatory cytokine production by inhibiting COX-2

ACE, angiotensin-converting enzyme; ARBs, angiotensin receptor blockers; COX-2, cyclooxygenase 2; IL, interleukin; PTSD, post-traumatic stress disorder; SSRIs, selective serotonin reuptake inhibitors; TNF-α, tumor necrosis factor-α.

## Data Availability

No new data were created or analyzed in this study. Data sharing is not applicable to this article.

## References

[1] Stein D.J., McLaughlin K.A., Koenen K.C., Atwoli L., Friedman M.J., Hill E.D., Maercker A., Petukhova M., Shahly V., van Ommeren M. (2014). DSM-5 and ICD-11 Definitions of Posttraumatic Stress Disorder: Investigating “Narrow” and “Broad” Approaches. Depress. Anxiety.

[2] Rosenfield P.J., Stratyner A., Tufekcioglu S., Karabell S., Mckelvey J., Litt L. (2018). Complex PTSD in ICD-11: A Case Report on a New Diagnosis. J. Psychiatr. Pract..

[3] Iribarren J., Prolo P., Neagos N., Chiappelli F. (2005). Post-Traumatic Stress Disorder: Evidence-Based Research for the Third Millennium. Evid. Based Complement. Alternat. Med..

[4] Brewin C.R. (2001). A Cognitive Neuroscience Account of Posttraumatic Stress Disorder and its Treatment. Behav. Res. Ther..

[5] McNally R.J. (2006). Cognitive Abnormalities in Post-Traumatic Stress Disorder. Trends Cogn. Sci..

[6] Ehlers A., Clark D.M. (2000). A Cognitive Model of Posttraumatic Stress Disorder. Behav. Res. Ther..

[7] Koenen K.C., Ratanatharathorn A., Ng L., McLaughlin K.A., Bromet E.J., Stein D.J., Karam E.G., Ruscio A.M., Benjet C., Scott K. (2017). Posttraumatic Stress Disorder in the World Mental Health Surveys. Psychol. Med..

[8] Kessler R.C., Berglund P., Demler O., Jin R., Merikangas K.R., Walters E.E. (2005). Lifetime Prevalence and Age-of-Onset Distributions of DSM-IV Disorders in the National Comorbidity Survey Replication. Arch. Gen. Psychiatry.

[9] Kang H.K., Natelson B.H., Mahan C.M., Lee K.Y., Murphy F.M. (2003). Post-Traumatic Stress Disorder and Chronic Fatigue Syndrome-Like Illness Among Gulf War Veterans: A Population-Based Survey of 30,000 Veterans. Am. J. Epidemiol..

[10] Eraly S.A., Nievergelt C.M., Maihofer A.X., Barkauskas D.A., Biswas N., Agorastos A., O’Connor D.T., Baker D.G., Marine Resiliency Study Team (2014). Assessment of Plasma C-Reactive Protein as a Biomarker of Posttraumatic Stress Disorder Risk. JAMA Psychiatry.

[11] Cohen M., Meir T., Klein E., Volpin G., Assaf M., Pollack S. (2011). Cytokine Levels as Potential Biomarkers for Predicting the Development of Posttraumatic Stress Symptoms in Casualties of Accidents. Int. J. Psychiatry Med..

[12] Wang Z., Young M.R.I. (2016). PTSD, a Disorder with an Immunological Component. Front. Immunol..

[13] Pace T.W., Heim C.M. (2011). A Short Review on the Psychoneuroimmunology of Posttraumatic Stress Disorder: From Risk Factors to Medical Comorbidities. Brain Behav. Immun..

[14] Olff M., van Zuiden M. (2017). Neuroendocrine and Neuroimmune Markers in PTSD: Pre-, Peri- and Post-Trauma Glucocorticoid and Inflammatory Dysregulation. Curr. Opin. Psychol..

[15] Lopresti A.L., Drummond P.D. (2013). Obesity and Psychiatric Disorders: Commonalities in Dysregulated Biological Pathways and their Implications for Treatment. Prog. Neuropsychopharmacol. Biol. Psychiatry.

[16] Levine A.B., Levine L.M., Levine T.B. (2014). Posttraumatic Stress Disorder and Cardiometabolic Disease. Cardiology.

[17] O’Donovan A., Cohen B.E., Seal K.H., Bertenthal D., Margaretten M., Nishimi K., Neylan T.C. (2015). Elevated Risk for Autoimmune Disorders in Iraq and Afghanistan Veterans with Posttraumatic Stress Disorder. Biol. Psychiatry.

[18] Banks W.A., Kastin A.J., Broadwell R.D. (1995). Passage of Cytokines Across the Blood-Brain Barrier. Neuroimmunomodulation.

[19] Liu B., Hong J.S. (2003). Role of Microglia in Inflammation-Mediated Neurodegenerative Diseases: Mechanisms and Strategies for Therapeutic Intervention. J. Pharmacol. Exp. Ther..

[20] de Pablos R.M., Herrera A.J., Espinosa-Oliva A.M., Sarmiento M., Muñoz M.F., Machado A., Venero J.L. (2014). Chronic Stress Enhances Microglia Activation and Exacerbates Death of Nigral Dopaminergic Neurons Under Conditions of Inflammation. J. Neuroinflamm..

[21] Baker D.G., Ekhator N.N., Kasckow J.W., Hill K.K., Zoumakis E., Dashevsky B.A., Chrousos G.P., Geracioti T.D. (2001). Plasma and Cerebrospinal Fluid Interleukin-6 Concentrations in Posttraumatic Stress Disorder. Neuroimmunomodulation.

[22] Bonne O., Gill J.M., Luckenbaugh D.A., Collins C., Owens M.J., Alesci S., Neumeister A., Yuan P., Kinkead B., Manji H.K. (2011). Corticotropin-Releasing Factor, Interleukin-6, Brain-Derived Neurotrophic Factor, Insulin-Like Growth Factor-1, and Substance P in the Cerebrospinal Fluid of Civilians with Posttraumatic Stress Disorder Before and After Treatment with Paroxetine. J. Clin. Psychiatry.

[23] Amos T., Stein D.J., Ipser J.C. (2014). Pharmacological Interventions for Preventing Post-Traumatic Stress Disorder (PTSD). Cochrane Database Syst. Rev..

[24] Birur B., Math S.B., Fargason R.E. (2017). A Review of Psychopharmacological Interventions Post-Disaster to Prevent Psychiatric Sequelae. Psychopharmacol. Bull..

[25] Ferrero-Miliani L., Nielsen O.H., Andersen P.S., Girardin S. (2007). Chronic Inflammation: Importance of NOD2 and NALP3 in Interleukin-1beta Generation. Clin. Exp. Immunol..

[26] Dantzer R., O’connor J.C., Freund G.G., Johnson R.W., Kelley K.W. (2008). From Inflammation to Sickness and Depression: When the Immune System Subjugates the Brain. Nat. Rev. Neurosci..

[27] Levin S.G., Godukhin O.V. (2017). Modulating Effect of Cytokines on Mechanisms of Synaptic Plasticity in the Brain. Biochemistry.

[28] Butovsky O., Weiner H.L. (2018). Microglial Signatures and their Role in Health and Disease. Nat. Rev. Neurosci..

[29] Reus G.Z., Fries G.R., Stertz L., Badawy M., Passos I.C., Barichello T., Kapczinski F., Quevedo J. (2015). The Role of Inflammation and Microglial Activation in the Pathophysiology of Psychiatric Disorders. Neuroscience.

[30] Takaki J., Fujimori K., Miura M., Suzuki T., Sekino Y., Sato K. (2012). L-Glutamate Released from Activated Microglia Downregulates Astrocytic L-Glutamate Transporter Expression in Neuroinflammation: The ‘Collusion’ Hypothesis for Increased Extracellular L-Glutamate Concentration in Neuroinflammation. J. Neuroinflamm..

[31] Mellon S.H., Gautam A., Hammamieh R., Jett M., Wolkowitz O.M. (2018). Metabolism, Metabolomics, and Inflammation in Posttraumatic Stress Disorder. Biol. Psychiatry.

[32] Wang Z., Mandel H., Levingston C.A., Young M.R.I. (2016). An Exploratory Approach Demonstrating Immune Skewing and a Loss of Coordination Among Cytokines in Plasma and Saliva of Veterans with Combat-Related PTSD. Hum. Immunol..

[33] Hori H., Kim Y. (2019). Inflammation and Post-Traumatic Stress Disorder. Psychiatry Clin. Neurosci..

[34] Imai R., Hori H., Itoh M., Lin M., Niwa M., Ino K., Ogawa S., Ishida M., Sekiguchi A., Matsui M. (2018). Inflammatory Markers and their Possible Effects on Cognitive Function in Women with Posttraumatic Stress Disorder. J. Psychiatr. Res..

[35] Lindqvist D., Dhabhar F.S., Mellon S.H., Yehuda R., Grenon S.M., Flory J.D., Bierer L.M., Abu-Amara D., Coy M., Makotkine I. (2017). Increased Pro-Inflammatory Milieu in Combat Related PTSD—A New Cohort Replication Study. Brain Behav. Immun..

[36] De Oliveira J.F., Wiener C.D., Jansen K., Portela L.V., Lara D.R., de Mattos Souza L.D., da Silva R.A., Moreira F.P., Oses J.P. (2018). Serum Levels of Interleukins IL-6 and IL-10 in Individuals with Posttraumatic Stress Disorder in a Population-Based Sample. Psychiatry Res..

[37] Rohleder N., Joksimovic L., Wolf J.M., Kirschbaum C. (2004). Hypocortisolism and Increased Glucocorticoid Sensitivity of Pro-Inflammatory Cytokine Production in Bosnian War Refugees with Posttraumatic Stress Disorder. Biol. Psychiatry.

[38] Gill J., Vythilingam M., Page G.G. (2008). Low Cortisol, High DHEA, and High Levels of Stimulated TNF-Alpha, and IL-6 in Women with PTSD. J. Trauma Stress.

[39] Bruenig D., Mehta D., Morris C.P., Harvey W., Lawford B., Young R.M., Voisey J. (2017). Genetic and Serum Biomarker Evidence for a Relationship Between TNFalpha and PTSD in Vietnam War Combat Veterans. Compr. Psychiatry.

[40] O’Donovan A., Ahmadian A.J., Neylan T.C., Pacult M.A., Edmondson D., Cohen B.E. (2017). Current Posttraumatic Stress Disorder and Exaggerated Threat Sensitivity Associated with Elevated Inflammation in the Mind Your Heart Study. Brain Behav. Immun..

[41] Miller M.W., Maniates H., Wolf E.J., Logue M.W., Schichman S.A., Stone A., Milberg W., McGlinchey R. (2018). CRP Polymorphisms and DNA Methylation of the AIM2 Gene Influence Associations Between Trauma Exposure, PTSD, and C-Reactive Protein. Brain Behav. Immun..

[42] Wang Z., Caughron B., Young M.R.I. (2017). Posttraumatic Stress Disorder: An Immunological Disorder?. Front. Psychiatry.

[43] Lindqvist D., Mellon S.H., Dhabhar F.S., Yehuda R., Grenon S.M., Flory J.D., Bierer L.M., Abu-Amara D., Coy M., Makotkine I. (2017). Increased Circulating Blood Cell Counts in Combat-Related PTSD: Associations with Inflammation and PTSD Severity. Psychiatry Res..

[44] Bersani F.S., Wolkowitz O.M., Milush J.M., Sinclair E., Eppling L., Aschbacher K., Lindqvist D., Yehuda R., Flory J., Bierer L.M. (2016). A Population of Atypical CD56(−)CD16(+) Natural Killer Cells is Expanded in PTSD and is Associated with Symptom Severity. Brain Behav. Immun..

[45] Gill J., Luckenbaugh D., Charney D., Vythilingam M. (2010). Sustained Elevation of Serum Interleukin-6 and Relative Insensitivity to Hydrocortisone Differentiates Posttraumatic Stress Disorder With and Without Depression. Biol. Psychiatry.

[46] Passos I.C., Vasconcelos-Moreno M.P., Costa L.G., Kunz M., Brietzke E., Quevedo J., Salum G., Magalhães P.V., Kapczinski F., Kauer-Sant’Anna M. (2015). Inflammatory Markers in Post-Traumatic Stress Disorder: A Systematic Review, Meta-Analysis, and Meta-Regression. Lancet Psychiatry.

[47] Lindqvist D., Wolkowitz O.M., Mellon S., Yehuda R., Flory J.D., Henn-Haase C., Bierer L.M., Abu-Amara D., Coy M., Neylan T.C. (2014). Proinflammatory Milieu in Combat-Related PTSD is Independent of Depression and Early Life Stress. Brain Behav. Immun..

[48] Von Kanel R., Hepp U., Kraemer B., Traber R., Keel M., Mica L., Schnyder U. (2007). Evidence for Low-Grade Systemic Proinflammatory Activity in Patients with Posttraumatic Stress Disorder. J. Psychiatr. Res..

[49] Gola H., Engler H., Sommershof A., Adenauer H., Kolassa S., Schedlowski M., Groettrup M., Elbert T., Kolassa I.T. (2013). Posttraumatic Stress Disorder is Associated with an Enhanced Spontaneous Production of Pro-Inflammatory Cytokines by Peripheral Blood Mononuclear Cells. BMC Psychiatry.

[50] Koolhaas J.M., Korte S.M., de Boer S.F., van der Vegt B.J., van Reenen C.G., Hopster H., de Jong I.C., Ruis M.A.W., Blokhuis H.J. (1999). Coping Styles in Animals: Current Status in Behavior and Stress-Physiology. Neurosci. Biobehav. Rev..

[51] Koolhaas J.M., de Boer S.F., Buwalda B., van Reenen K. (2007). Individual Variation in Coping with Stress: A Multidimensional Approach of Ultimate and Proximate Mechanisms. Brain Behav. Evol..

[52] Sih A., Bell A.M., Johnson J.C., Ziemba R.E. (2004). Behavioral Syndromes: An Intergrative Overiew. Q. Rev. Biol..

[53] Bell A.M. (2007). Evolutionary Biology: Animal Personalities. Nature.

[54] Reale D., Reader S.M., Sol D., McDougall P.T., Dingemanse N.J. (2007). Integrating Animal Temperament Within Ecology and Evolution. Biol. Rev. Camb. Philos. Soc..

[55] Borghans B., Homberg J.R. (2015). Animal Models for Posttraumatic Stress Disorder: An Overview of What is Used in Research. World J. Psychiatry.

[56] Schoner J., Heinz A., Endres M., Gertz K., Kronenberg G. (2017). Post-Traumatic Stress Disorder and Beyond: An Overview of Rodent Stress Models. J. Cell. Mol. Med..

[57] Deslauriers J., Toth M., Der-Avakian A., Risbrough V.B. (2018). Current Status of Animal Models of Posttraumatic Stress Disorder: Behavioral and Biological Phenotypes, and Future Challenges in Improving Translation. Biol. Psychiatry.

[58] Liberzon I., Krstov M., Young E.A. (1997). Stress-Restress: Effects on ACTH and Fast Feedback. Psychoneuroendocrinology.

[59] Liberzon I., Lopez J.F., Flagel S.B., Vazquez D.M., Young E.A. (1999). Differential Regulation of Hippocampal Glucocorticoid Receptors mRNA and Fast Feedback: Relevance to Post-Traumatic stress disorder. J. Neuroendocrinol..

[60] Van Dijken H.H., van der Heyden J.A., Mos J., Tilders F.J. (1992). Inescapable Footshocks Induce Progressive and Long-Lasting Behavioural Changes in Male Rats. Physiol. Behav..

[61] Debiec J., Bush D.E., LeDoux J.E. (2011). Noradrenergic Enhancement of Reconsolidation in the Amygdala Impairs Extinction of Conditioned Fear in Rats--A Possible Mechanism for the Persistence of Traumatic Memories in PTSD. Depress. Anxiety.

[62] Belda X., Rotllant D., Fuentes S., Delgado R., Nadal R., Armario A. (2008). Exposure to Severe Stressors Causes Long-Lasting Dysregulation of Resting and Stress-Induced Activation of the Hypothalamic-Pituitary-Adrenal Axis. Ann. N. Y. Acad. Sci..

[63] Daskalakis N.P., Lehrner A., Yehuda R. (2013). Endocrine Aspects of Post-Traumatic Stress Disorder and Implications for Diagnosis and Treatment. Endocrinol. Metab. Clin. N. Am..

[64] Zoladz P.R., Conrad C.D., Fleshner M., Diamond D.M. (2008). Acute Episodes of Predator Exposure in Conjunction with Chronic Social Instability as an Animal Model of Post-Traumatic Stress Disorder. Stress.

[65] Zoladz P.R., Fleshner M., Diamond D.M. (2012). Psychosocial Animal Model of PTSD Produces a Long-Lasting Traumatic Memory, an Increase in General Anxiety and PTSD-Like Glucocorticoid Abnormalities. Psychoneuroendocrinology.

[66] Pibiri F., Nelson M., Guidotti A., Costa E., Pinna G. (2008). Decreased Corticolimbic Allopregnanolone Expression During Social Isolation Enhances Contextual Fear: A Model Relevant for Posttraumatic Stress Disorder. Proc. Natl. Acad. Sci. USA.

[67] de Jongh R., Geyer M.A., Olivier B., Groenink L. (2005). The Effects of Sex and Neonatal Maternal Separation on Fear-Potentiated and Light-Enhanced Startle. Behav. Brain Res..

[68] Yehuda R. (2009). Status of Glucocorticoid Alterations in Post-Traumatic Stress Disorder. Ann. N. Y. Acad. Sci..

[69] Cohen H., Kaplan Z., Matar M.A., Loewenthal U., Zohar J., Richter-Levin G. (2007). Long-Lasting Behavioral Effects of Juvenile Trauma in an Animal Model of PTSD Associated with a Failure of the Autonomic Nervous System to Recover. Eur. Neuropsychopharmacol..

[70] Wilson C.B., Ebenezer P.J., McLaughlin L.D., Francis J. (2014). Predator Exposure/Psychosocial Stress Animal Model of Post-Traumatic Stress Disorder Modulates Neurotransmitters in the Rat Hippocampus and Prefrontal Cortex. PLoS ONE.

[71] Deslauriers J., Powell S., Risbrough V.B. (2017). Immune Signaling Mechanisms of PTSD Risk and Symptom Development: Insights from Animal Models. Curr. Opin. Behav. Sci..

[72] Jones M.E., Lebonville C.L., Barrus D., Lysle D.T. (2015). The Role of Brain Interleukin-1 in Stress-Enhanced Fear Learning. Neuropsychopharmacology.

[73] Zimmerman G., Shaltiel G., Barbash S., Cohen J., Gasho C.J., Shenhar-Tsarfaty S., Shalev H., Berliner S.A., Shelef I., Shoham S. (2012). Post-Traumatic Anxiety Associates with Failure of the Innate Immune Receptor TLR9 to Evade the Pro-Inflammatory NFkappaB Pathway. Transl. Psychiatry.

[74] Muhie S., Gautam A., Chakraborty N., Hoke A., Meyerhoff J., Hammamieh R., Jett M. (2017). Molecular Indicators of Stress-Induced Neuroinflammation in a Mouse Model Simulating Features of Post-Traumatic Stress Disorder. Transl. Psychiatry.

[75] Korte S.M., Buwalda B., Bouws G.A.H., Koolhaas J.M., Maes F.W., Bohus B. (1992). Conditioned Neuroendocrine and Cardiovascular Stress Responsiveness Accompanying Behavioral Passivity and Activity in Aged and in Young Rats. Physiol. Behav..

[76] Perez-Tejada J., Arregi A., Gomez-Lazaro E., Vegas O., Azpiroz A., Garmendia L. (2013). Coping with Chronic Social Stress in Mice: Hypothalamic-Pituitary-Adrenal/Sympathetic-Adrenal-Medullary Axis Activity, Behavioral Changes and Effects of Antalarmin Treatment: Implications for the Study of Stress-Related Psychopathologies. Neuroendocrinology.

[77] Wood S.K., Walker H.E., Valentino R.J., Bhatnagar S. (2010). Individual Differences in Reactivity to Social Stress Predict Susceptibility and Resilience to a Depressive Phenotype: Role of Corticotropin-Releasing Factor. Endocrinology.

[78] De Miguel Z., Vegas O., Garmendia L., Arregi A., Beitia G., Azpiroz A. (2011). Behavioral Coping Strategies in Response to Social Stress are Associated with Distinct Neuroendocrine, Monoaminergic and Immune Response Profiles in Mice. Behav. Brain Res..

[79] Gomez-Lazaro E., Arregi A., Beitia G., Vegas O., Azpiroz A., Garmendia L. (2011). Individual Differences in Chronically Defeated Male Mice: Behavioral, Endocrine, Immune, and Neurotrophic Changes as Markers of Vulnerability to the Effects of Stress. Stress.

[80] Wohleb E.S., Fenn A.M., Pacenta A.M., Powell N.D., Sheridan J.F., Godbout J.P. (2012). Peripheral Innate Immune Challenge Exaggerated Microglia Activation, Increased the Number of Inflammatory CNS Macrophages, and Prolonged Social Withdrawal in Socially Defeated Mice. Psychoneuroendocrinology.

[81] Wohleb E.S., Patterson J.M., Sharma V., Quan N., Godbout J.P., Sheridan J.F. (2014). Knockdown of Interleukin-1 Receptor Type-1 on Endothelial Cells Attenuated Stress-Induced Neuroinflammation and Prevented Anxiety-Like Behavior. J. Neurosci..

[82] Powell N.D., Sloan E.K., Bailey M.T., Arevalo J.M., Miller G.E., Chen E., Kobor M.S., Reader B.F., Sheridan J.F., Cole S.W. (2013). Social Stress Up-Regulates Inflammatory Gene Expression in the Leukocyte Transcriptome via Beta-Adrenergic Induction of Myelopoiesis. Proc. Natl. Acad. Sci. USA.

[83] Wood S.K., Wood C.S., Lombard C.M., Lee C.S., Zhang X.Y., Finnell J.E., Valentino R.J. (2015). Inflammatory Factors Mediate Vulnerability to a Social Stress-Induced Depressive-like Phenotype in Passive Coping Rats. Biol. Psychiatry.

[84] Jones K.A., Thomsen C. (2013). The role of the innate immune system in psychiatric disorders. Mol. Cell. Neurosci..

[85] Frank M.G., Weber M.D., Watkins L.R., Maier S.F. (2016). Stress-induced neuroinflammatory priming: A liability factor in the etiology of psychiatric disorders. Neurobiol. Stress.

[86] Albus C. (2010). Psychological and social factors in coronary heart disease. Ann. Med..

[87] Sansone R.A., Sansone L.A. (2008). Depression and cardiovascular disease: Just an urban legend?. Psychiatry.

[88] Schroder K., Tschopp J. (2010). The inflammasomes. Cell.

[89] Kumar V. (2019). Toll-like receptors in the pathogenesis of neuroinflammation. J. Neuroimmunol..

[90] Heneka M.T., Kummer M.P., Latz E. (2014). Innate immune activation in neurodegenerative disease. Nat. Rev. Immunol..

[91] Ransohoff R.M. (2016). How neuroinflammation contributes to neurodegeneration. Science.

[92] Daskalakis N.P., Cohen H., Nievergelt C.M., Baker D.G., Buxbaum J.D., Russo S.J., Yehuda R. (2016). New Translational Perspectives for Blood-Based Biomarkers of PTSD: From Glucocorticoid to Immune Mediators of Stress Susceptibility. Exp. Neurol..

[93] Hendrickson R.C., Raskind M.A. (2016). Noradrenergic Dysregulation in the Pathophysiology of PTSD. Exp. Neurol..

[94] ter Heegde F., De Rijk R.H., Vinkers C.H. (2015). The Brain Mineralocorticoid Receptor and Stress Resilience. Psychoneuroendocrinology.

[95] Berger I., Werdermann M., Bornstein S.R., Steenblock C. (2019). The adrenal gland in stress—Adaptation on a cellular level. J. Steroid Biochem. Mol. Biol..

[96] Kim Y.K., Amidfar M., Won E. (2019). A Review on Inflammatory Cytokine-Induced Alterations of the Brain as Potential Neural Biomarkers in Post-Traumatic Stress Disorder. Prog. Neuropsychopharmacol. Biol. Psychiatry.

[97] Sarapultsev A., Sarapultsev P., Dremencov E., Komelkova M., Tseilikman O., Tseilikman V. (2020). Low glucocorticoids in stress-related disorders: The role of inflammation. Stress.

[98] Pariante C.M. (2017). Why are depressed patients inflamed? A reflection on 20 years of research on depression, glucocorticoid resistance and inflammation. Eur. Neuropsychopharmacol..

[99] Yirmiya R., Goshen I. (2011). Immune modulation of learning, memory, neural plasticity and neurogenesis. Brain Behav. Immun..

[100] Ban E., Milon G., Prudhomme N., Fillion G., Haour F. (1991). Receptors for interleukin-1 (alpha and beta) in mouse brain: Mapping and neuronal localization in hippocampus. Neuroscience.

[101] Wong M.L., Licinio J. (1994). Localization of interleukin 1 type I receptor mRNA in rat brain. Neuroimmunomodulation.

[102] Hammond E.A., Smart D., Toulmond S., Suman-Chauhan N., Hughes J., Hall M.D. (1999). The interleukin-1 type I receptor is expressed in human hypothalamus. Brain.

[103] Myint A.M., Schwarz M.J., Muller N. (2012). The Role of the Kynurenine Metabolism in Major Depression. J. Neural Transm..

[104] Dostal C.R., Gamsby N.S., Lawson M.A., McCusker R.H. (2018). Glia- and Tissue-Specific Changes in the Kynurenine Pathway after Treatment of Mice with Lipopolysaccharide and Dexamethasone. Brain Behav. Immun..

[105] Gamble-George J.C., Baldi R., Halladay L., Kocharian A., Hartley N., Silva C.G., Roberts H., Haymer A., Marnett L.J., Holmes A. (2016). Cyclooxygenase-2 Inhibition Reduces Stress-Induced Affective Pathology. eLife.

[106] Kao C.Y., He Z., Henes K., Asara J.M., Webhofer C., Filiou M.D., Khaitovich P., Wotjak C.T., Turck C.W. (2016). Fluoxetine Treatment Rescues Energy Metabolism Pathway Alterations in a Posttraumatic Stress Disorder Mouse Model. Mol. Neuropsychiatry.

[107] Kao C.Y., He Z., Zannas A.S., Hahn O., Kühne C., Reichel J.M., Binder E.B., Wotjak C.T., Khaitovich P., Turck C.W. (2016). Fluoxetine Treatment Prevents the Inflammatory Response in a Mouse Model of Posttraumatic Stress Disorder. J. Psychiatr. Res..

[108] Lee B., Sur B., Yeom M., Shim I., Lee H., Hahm D.H. (2016). Effects of Systemic Administration of Ibuprofen on Stress Response in a Rat Model of Post-Traumatic Stress Disorder. Korean J. Physiol. Pharmacol..

[109] Levkovitz Y., Fenchel D., Kaplan Z., Zohar J., Cohen H. (2015). Early Post-Stressor Intervention with Minocycline, A Second-Generation Tetracycline, Attenuates Post-Traumatic Stress Response in an Animal Model of PTSD. Eur. Neuropsychopharmacol..

[110] Deslauriers J., van Wijngaarde M., Geyer M.A., Powell S., Risbrough V.B. (2017). Effects of LPS-Induced Immune Activation Prior to Trauma Exposure on PTSD-Like Symptoms in Mice. Behav. Brain Res..

[111] Ebenezer P.J., Wilson C.B., Wilson L.D., Nair A.R. (2016). The Anti-Inflammatory Effects of Blueberries in an Animal Model of Post-Traumatic Stress Disorder (PTSD). PLoS ONE.

[112] Kortekaas K.E., Meijer C.A., Hinnen J.W., Dalman R.L., Xu B., Hamming J.F., Lindeman J.H. (2014). ACE Inhibitors Potently Reduce Vascular Inflammation, Results of an Open Proof-of-Concept Study in the Abdominal Aortic Aneurysm. PLoS ONE.

[113] Clancy P., Koblar S.A., Golledge J. (2014). Angiotensin Receptor 1 Blockade Reduces Secretion of Inflammation Associated Cytokines from Cultured Human Carotid Atheroma and Vascular Cells in Association with Reduced Extracellular Signal Regulated Kinase Expression and Activation. Atherosclerosis.

[114] Quinones M.M., Maldonado L., Velazquez B., Porter J.T. (2016). Candesartan Ameliorates Impaired Fear Extinction Induced by Innate Immune Activation. Brain Behav. Immun..

[115] Aerni A., Traber R., Hock C., Roozendaal B., Schelling G., Papassotiropoulos A., Nitsch R.M., Schnyder U., de Quervain D.J.F. (2004). Low-Dose Cortisol for Symptoms of Posttraumatic Stress Disorder. Am. J. Psychiatry.

[116] Suris A., North C., Adinoff B., Powell C.M., Greene R. (2010). Effects of Exogenous Glucocorticoid on Combat-Related PTSD Symptoms. Ann. Clin. Psychiatry.

[117] Yehuda R., Bierer L.M., Pratchett L.C., Lehrner A., Koch E.C., Van Manen J.A., Flory J.D., Makotkine I., Hildebrandt T. (2015). Cortisol Augmentation of a Psychological Treatment for Warfighters with Posttraumatic Stress disorder: Randomized Trial Showing Improved Treatment Retention and Outcome. Psychoneuroendocrinology.

[118] Trenado C., Pedroarena-Leal N., Cif L., Nitsche M., Ruge D. (2018). Neural Oscillatory Correlates for Conditioning and Extinction of Fear. Biomedicines.

[119] Noble L.J., Gonzalez I.J., Meruva V.B., Callahan K.A., Belfort B.D., Ramanathan K.R., Meyers E., Kilgard M.P., Rennaker R.L., McIntyre C.K. (2017). Effects of vagus nerve stimulation on extinction of conditioned fear and post-traumatic stress disorder symptoms in rats. Transl. Psychiatry.

